# Mid-term safety and feasibility of an absorbable gelatin film adhesion barrier after laparoscopic colorectal resection

**DOI:** 10.1097/MS9.0000000000005325

**Published:** 2026-07-14

**Authors:** Hiroyuki Negishi, Masanori Naito, Kenta Katsumata, Hiro Nishizawa, Yuki Amano, Sota Usui, Hiroshi Nakano, Takehito Otsubo, Shinya Mikami

**Affiliations:** a Department of Gastroenterological and General Surgery, St. Marianna University Yokohama Seibu Hospital, Yokohama, Japan; b Department of Gastroenterological and General Surgery, St. Marianna University School of Medicine, Kawasaki, Japan

**Keywords:** absorbable gelatin film, adhesion barrier, laparoscopic colorectal surgery, postoperative adhesions, small bowel obstruction

## Abstract

**Background:**

With the current increase in life expectancy, the likelihood of undergoing multiple abdominal surgeries has risen. Adhesions from previous operations can prolong operative time, increase the risk of organ injury, and cause adhesive small bowel obstruction.

**Materials and methods:**

We evaluated the mid-term safety and feasibility of an absorbable gelatin film adhesion barrier (TENALEAF^®^ [AGF]; Medical Division, Gunze Limited, Tokyo, Japan) in laparoscopic colorectal resection for malignant tumors. Among 173 patients operated on between March 2022 and December 2023, 150 who received AGF placement beneath the umbilical incision into the peritoneal cavity were analyzed.

**Results:**

Tumor sites were the right colon in 48 cases, the left colon in 55, and the rectum in 47. The median operative time was 189.5 minutes (range: 95–544), and blood loss was 5 mL (range: 5–340). AGF was applied successfully in all cases. Postoperative complications included two superficial surgical site infections, one intra-abdominal abscess, one anastomotic leakage, and three cases of paralytic ileus. No adhesive bowel obstruction or AGF-related adverse events were observed during follow-up exceeding 1 year.

**Conclusion:**

AGF demonstrated safety and feasibility in preventing postoperative adhesions after laparoscopic colorectal resection. Further follow-up and case accumulation will be required to confirm long-term benefits.

## Introduction

With the global increase in life expectancy, the likelihood of undergoing multiple operations during a lifetime has risen. Postoperative adhesions frequently occur after abdominal surgery, with an incidence exceeding 90% after open procedures and 35.6–60% after laparoscopic surgery^[^1–3^].^ Adhesions are also recognized as causes of bowel obstruction, chronic abdominal pain, and infertility[4]. A systematic review estimated the incidence of postoperative adhesions observed during second-look surgery at 66% after gastrointestinal surgery, 51% after gynecologic surgery, and 22% after urologic surgery[5]. Adhesions from previous operations can prolong operative time and increase the risk of organ injury during reoperation.

HIGHLIGHTS

- An absorbable gelatin film (TENALEAF®) has recently been introduced in Japan, and several studies have reported its short-term outcomes.
- However, no previous investigations have evaluated its mid-term clinical safety and feasibility, particularly with follow-up exceeding 1 year.
- In 150 patients who underwent laparoscopic colorectal resection, no adhesive small bowel obstruction occurred, and no device-related adverse events were observed during a median follow-up of 676 days.
- An absorbable gelatin film may represent a safe and feasible option for reducing adhesion-related complications following colorectal surgery.

The incidence of adhesive small bowel obstruction resulting from intra-abdominal adhesions has been reported to be 65–75%[6]. The use of adhesion barriers during abdominal surgery has been shown to reduce the frequency, extent, and severity of adhesions; however, definitive evidence regarding their preventive effect on adhesive bowel obstruction remains limited^[^7,8^]^.

The absorbable gelatin film adhesion barrier, TENALEAF^®^ (AGF) (Medical Division, Gunze Limited, Tokyo, Japan), is a bioabsorbable anti-adhesion material with a textured gelatin surface. It remains at the application site as a gel for approximately 7 days to prevent adhesions and is enzymatically degraded within about 28 days[9]. Approved for clinical use in Japan in 2022, this material is a relatively new anti-adhesion barrier. In animal models, it showed superior physical strength and flexibility, as well as greater anti-adhesion efficacy compared with conventional hyaluronic acid-carboxymethylcellulose (HC) films, with no cytotoxicity[10]. However, clinical reports on its efficacy and safety are still limited. Although several studies have described short-term (3–6 months) outcomes^[^11,12^]^, there have been no reports evaluating mid- to long-term efficacy.

In this study, we evaluated the mid-term safety and feasibility of AGF in patients who underwent laparoscopic colorectal resection for colorectal cancer at our institution, with a follow-up period exceeding 1 year.

This cohort study has been reported in line with the STROCSS guidelines[13].

## Methods

This study was a descriptive, single-arm feasibility study conducted in accordance with the Declaration of Helsinki. Informed consent was obtained from all patients using the opt-out method. This retrospective, single-center cohort study was conducted at a tertiary care hospital.

**Figure 1.**
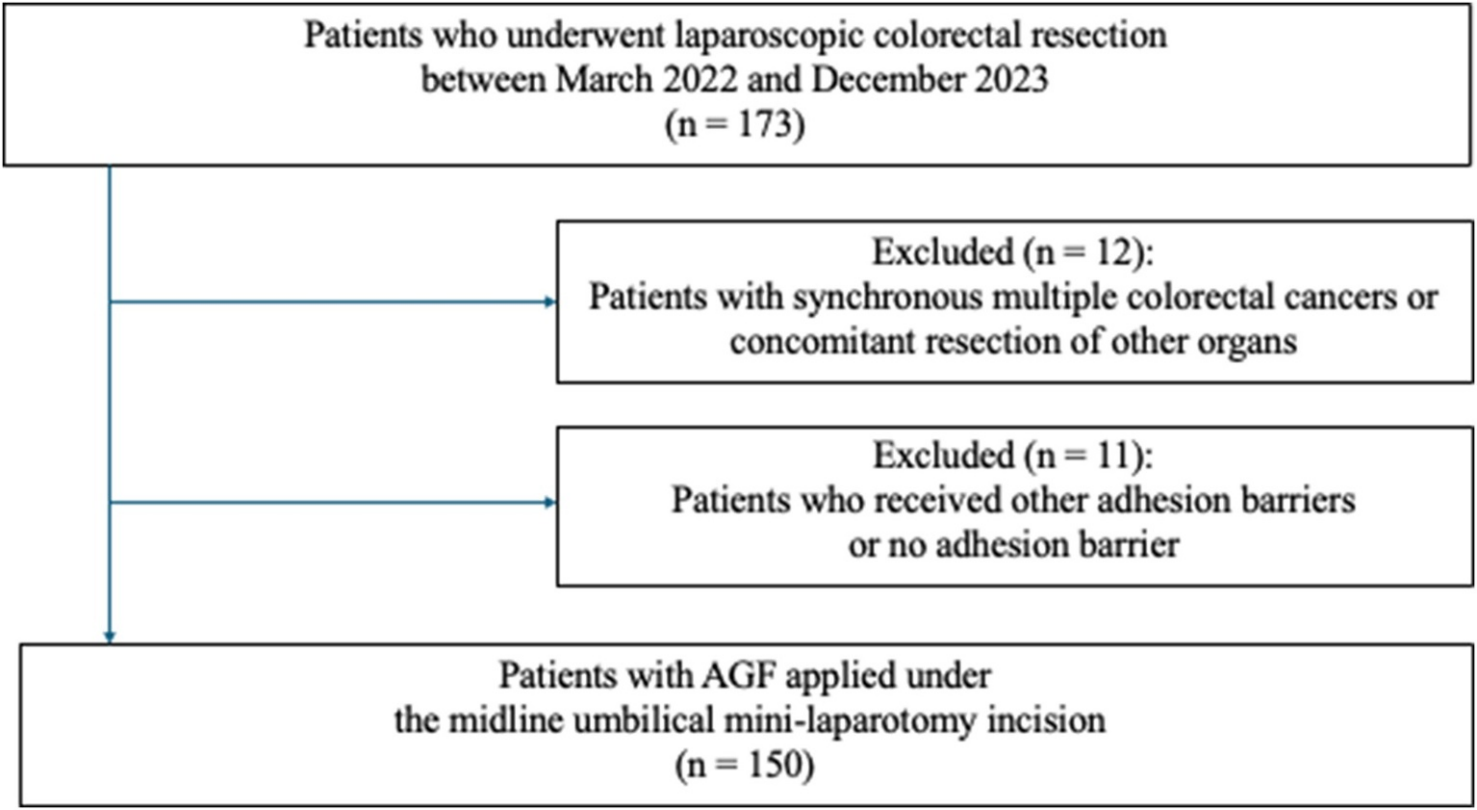
Patient selection flow diagram. AGF, absorbable gelatin film adhesion barrier (TENALEAF^®^; Medical Division, Gunze Limited, Tokyo, Japan)

Between March 2022 and December 2023, 173 patients underwent laparoscopic colorectal resection for colorectal cancer. Among these, 12 patients with multiple colorectal cancers or those who underwent concomitant resection of other organs were excluded. In addition, 11 patients who either received other types of adhesion barriers or no adhesion barrier were excluded. The remaining 150 patients, who received intraperitoneal placement of AGF beneath the umbilical mini-laparotomy incision at the time of abdominal closure, were included in this study (Fig. 1). The surgeon decided whether to use an anti-adhesive agent and, if so, which type to use.

**Figure 2.**
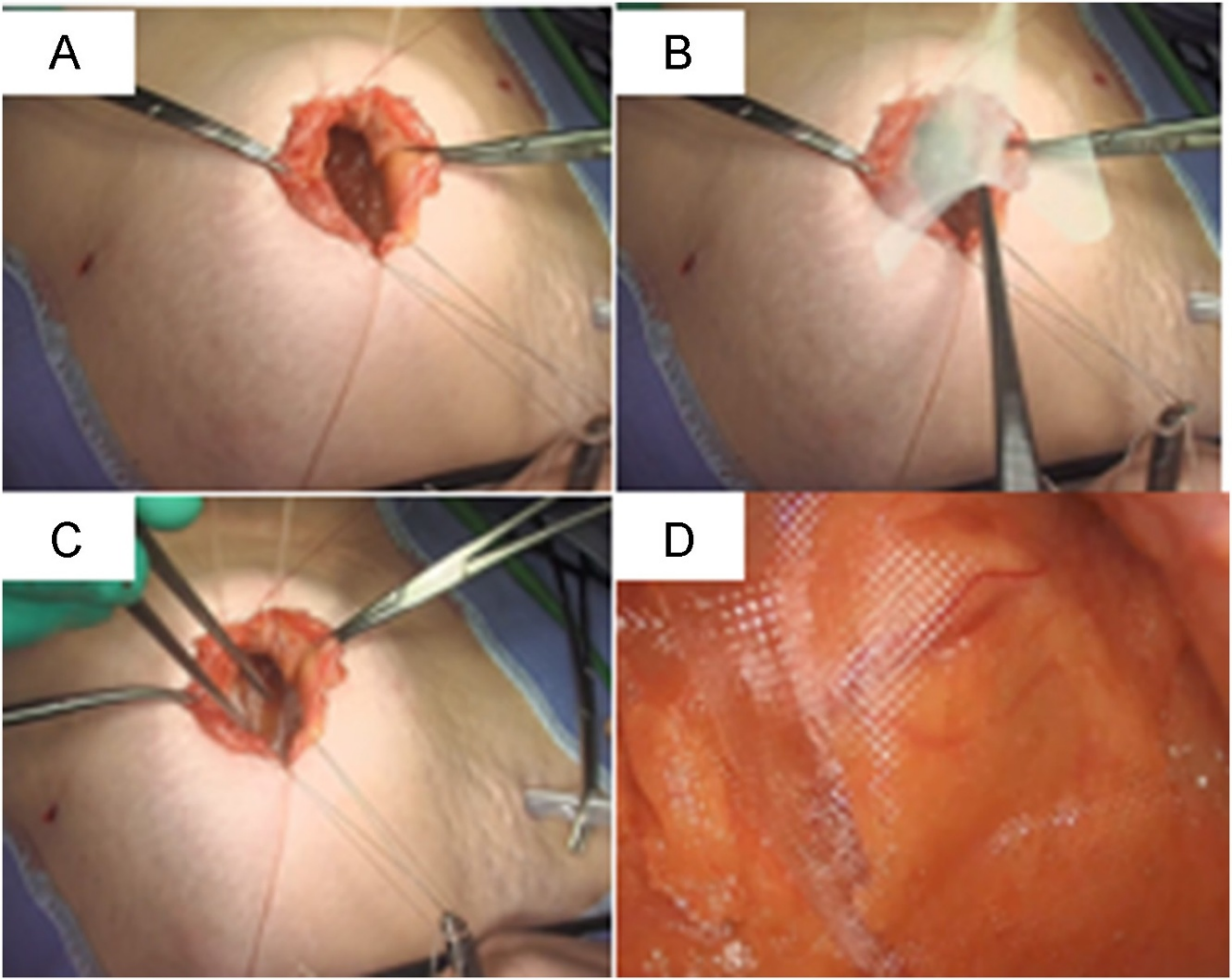
Intraoperative application of AGF. (A) Umbilical mini-laparotomy wound. (B) Insertion of AGF into the peritoneal cavity beneath the umbilical incision. (C) Position adjustment of AGF under the peritoneum. (D) Appearance after complete application within the abdominal cavity. AGF, absorbable gelatin film adhesion barrier (TENALEAF^®^; Medical Division, Gunze Limited, Tokyo, Japan).

Two sheets (7.3 × 6.3 cm each; size S) of AGF were used in each case. The sheets were applied beneath the umbilical incision into the peritoneal cavity during abdominal closure. The position was adjusted as necessary to ensure proper placement (Fig. 2).

The following variables were retrospectively evaluated: age, sex, postoperative complications, incidence of adhesive small bowel obstruction, and factors potentially related to adhesion formation or prevention^[^14,15^]^, including body mass index (BMI), surgical procedure, operative time, intraoperative blood loss, and umbilical incision length. Perioperative complications and the incidence of postoperative bowel obstruction were also assessed. Postoperative adhesions should ideally be evaluated by reoperation, but since this is an invasive procedure, we instead evaluated the incidence of adhesive bowel obstruction. The postoperative observation period extended until 31 December 2024.

Continuous variables are presented as median, minimum, and maximum values, and categorical variables are expressed as frequencies and percentages. The incidence of postoperative complications is reported with a 95% confidence interval (CI). Statistical analyses were performed using the Clopper-Pearson method with JMP^®^ Student Edition 18.2.1 (SAS Institute Inc., Cary, NC, USA).

## Results

The median age of the patients was 73.5 years (range: 23–92), and there were 81 men and 69 women. The median BMI was 22.3 kg/m^2^ (range: 13.0–36.4). Tumor locations included the right colon in 48 patients, the left colon in 55, and the rectum in 47. The surgical procedures consisted of right hemicolectomy in 45 patients, transverse colectomy in 8, left colectomy in 50, anterior resection in 43, abdominoperineal resection in 3, and Hartmann’s procedure in 1 (Table 1).

**Table 1:** Patient characteristics.

Variables	*n* = 150
Age (years)	73.5 (23−92)
Sex (male/female)	81 (54.0%)/69 (46.0%)
Body mass index (kg/m^2^)	22.3 (13.0–36.4)
Tumor location	48/55/47
Right side colon/Left side colon/Rectum	(32.0%/36.7%/31.3%)
Operation	45/8/50/43/3/1
RC/TC/LC/AR/APR/Hartmann	(30.0%/5.3%/33.3%/28.7%/2.0%/0.7%)

Data are presented as median (range) or number (%)

APR, abdominoperineal resection; AR, anterior resection; LC, left colectomy; TC, transverse colectomy; RC, right colectomy.

The median operative time was 189.5 minutes (range: 95–544), and the median blood loss was 5 mL (range: 5–340). The median umbilical incision length was 43.5 mm (range: 25–80). AGF was successfully applied in all cases without rupture or handling difficulties. The median postoperative observation period was 676 days (range: 370–1036).

Postoperative complications occurred in seven patients (4.6%) (Table 2). These included two cases of superficial surgical site infection (1.3%), one case of intra-abdominal abscess (0.7%), one case of anastomotic leakage (0.7%), and three cases of early postoperative paralytic ileus (2.0%). No adhesive small bowel obstruction was observed during the follow-up period (Table 3).

**Table 2:** Postoperative results.

Variables	*n* = 150
Operation time (min)	189.5 (95−544)
Estimated blood loss (ml)	5 (5−340)
Postoperative follow-up period (days)	676 (370−544)
Length of umbilicus small incision wound (mm)	43.5 (25−80)
Postoperative complications	7 (4.6%)

Data are presented as median (range) or number (%).

**Table 3:** Postoperative complications.

Number of subjects	*n* = 7	95% confidence interval
Superficial incisional surgical site infection	1.3% (2/150)	0.36−4.73%
Abdominal abscess	0.7% (1/150)	0.11−3.67%
Anastomotic leakage	0.7% (1/150)	0.11−3.67%
Paralytic ileus	2.0% (3/150)	0.68%−5.71%

Among the complications, only two (1.3%) were classified as Clavien–Dindo grade III or higher: one anastomotic leakage and one paralytic ileus. The AGF application site was anatomically distant from the anastomotic and abscess formation areas, and no adverse events attributable to AGF were identified.

## Discussion

Although definitive evidence supporting the preventive effect of adhesion barriers on adhesive bowel obstruction is limited, many studies have reported reductions in the frequency, extent, and severity of postoperative adhesions.^[^4,7,8,11,12,14–21^]^ Commonly used adhesion barriers include oxidized regenerated cellulose, HC, icodextrin solution, and polyethylene glycol gel. Meta-analyses comparing these agents with no treatment at all have shown no serious adverse events associated with any of the four agents[7]

Among them, HC has been widely used in abdominal surgery and has demonstrated efficacy in reducing postoperative adhesions. However, because it readily adheres to moist tissues and tears easily, its handling properties are suboptimal[18]. In contrast, as a relatively new agent approved for clinical use in Japan in March 2022, AGF provides adequate strength and flexibility, is resistant to tearing, and can be repositioned during application. Nevertheless, few clinical studies have evaluated its anti-adhesion efficacy and safety.

Watanabe *et al*[11] conducted a clinical study involving 123 patients with primary rectal cancer who underwent laparoscopic surgery with temporary loop ileostomy. During a mean follow-up of 6 months, the non-adhesion rate was 66.1% in the AGF group and 55.7% in the control group, indicating noninferiority[11]. Kikuchi *et al*[12] evaluated AGF use in 30 patients undergoing gynecologic surgery and reported no cases of adhesive bowel obstruction or AGF-related adverse events during a 12-week follow-up period.

In the present study, no adhesive small bowel obstruction was observed during a follow-up period exceeding 1 year. One intra-abdominal abscess and one anastomotic leakage occurred; however, these events were anatomically distant from the AGF application site and were not considered to be AGF-related.

Generally, evaluating postoperative adhesions using ultrasound, computed tomography, or magnetic resonance imaging is not easy. While detailed evaluation might be possible through second-look surgery, such evaluation is undesirably invasive. Therefore, adhesive bowel obstruction was evaluated as an alternative endpoint^[^12,14^]^.

This study had a relatively small sample size (*n* = 150) and a limited observation period, with a median follow-up of 676 days (range: 370–1036). While longer than that in previous reports, the duration remains insufficient for evaluating long-term outcomes. Fazio *et al*[15] estimated that a randomized controlled trial to assess the effect of adhesion barriers on bowel obstruction would require approximately 1700 cases, assuming a 20% incidence over 2 years. Furthermore, Fevang *et al*[21] followed 500 patients who underwent surgery for adhesive bowel obstruction and reported that the interval between the most recent abdominal operation and the first surgery for adhesive obstruction was within 1 year in 41%, within 1–5 years in 26%, within 5–10 years in 11%, and over 10 years in 22%. These findings suggest that evaluating the preventive effect of adhesion barriers on bowel obstruction requires multi-year follow-up.

Opinions remain divided in the field of postoperative adhesion prevention. Despite extensive research on various barrier materials, no definitive gold standard for adhesion prevention barriers has yet been established [22]. The mechanisms underlying adhesion formation are multifaceted, and current strategies are transitioning from simple physical barriers toward more biologically active interventions[23].

As demonstrated by recent large-scale real-world studies, retrospective cohort studies using claims databases are increasingly recognized as a vital approach to providing supplementary clinical evidence.[24] Our study is a foundational, descriptive cohort analysis that contributes to this body of real-world evidence.

In laparoscopic colorectal surgery, AGF demonstrated safety and feasibility, with no adhesion-related complications or device-associated adverse events observed during a follow-up period exceeding 1 year. This study had several limitations, including its retrospective design, the possibility of selection bias, a small sample size, a short follow-up period, the absence of a control group, and the lack of direct adhesion assessment. Nevertheless, our findings suggest that AGF may be a safe and feasible option for preventing postoperative adhesions following laparoscopic colorectal surgery. To confirm the efficacy and safety of AGF, further accumulation of cases and long-term observation are warranted, along with the performance of larger-scale, multi-institutional, randomized controlled trials with longer follow-up periods.

## Data Availability

The data supporting the findings of this study are available from the corresponding author upon reasonable request.

## Research registration unique identifying number (UIN)

This study was not registered with a clinical trial registry, as it was an observational cohort without an intervention.
